# Editorial: Molecular basis of epigenetic regulation in cancer therapies

**DOI:** 10.3389/fgene.2022.1115353

**Published:** 2023-01-10

**Authors:** Angeles Carlos-Reyes, Susana Romero-Garcia, César López-Camarillo, Guillermo Barreto, Heriberto Prado-Garcia

**Affiliations:** ^1^ Laboratorio de Onco-Inmunobiología, Departamento de Enfermedades Crónico-Degenerativas, Instituto Nacional de Enfermedades Respiratorias “Ismael Cosio Villegas”, Mexico City, Mexico; ^2^ Facultad de Ciencias, National University of Mexico, Mexico City, Mexico; ^3^ Posgrado en Ciencias Genómicas, Universidad Autónoma de la Ciudad de México, Ciudad de Mexico, Mexico; ^4^ Laboratoire IMoPA, UMR 7365 CNRS-Université de Lorraine, Biopôle de l'Université de Lorraine, Vandoeuvre-lès-Nancy Cedex, France

**Keywords:** chemoresistance, epigenetics, chemotherapy, bioinformatics, cancer hallmarks

## 1 Introduction

Cancer is not only a genetic disease, but it also comprehends epigenetic changes. Hanahan proposed non-mutational epigenetic reprogramming as an emerging cancer hallmark, applicable across multiple malignant tumors (Hanahan, 2022). As the reader can see in the articles contained in our research topic, non-mutational epigenetic programming is a common trait. As such, it promotes tumor development, is associated with a poor prognosis, and can be targeted for therapy. Castro-Oropeza and Pina-Sanchez comprehensively review the epigenetic alterations induced by human papillomavirus (HPV); conversely, Hurnik et al. review several epigenetic changes found in head and neck squamous cell carcinoma (HNSCC) (Hurnik et al.). Castro-Oropeza and Pina-Sanchez review focuses on alterations induced by HPV, while Hurnik’s centers on the external factors of tobacco and alcohol on the development of HNSCC (Castro-Oropeza and Pina-Sanchez; Hurnik et al.). Nevertheless, both reviews show that alterations in DNA methylation, histone modifications, and non-coding RNAs promote the progression of seemingly unrelated tumors.

## 2 Epigenetic regulation on cancer

Tumor heterogeneity is responsible for tumor cells possessing different sensibilities to environmental factors, growth, invasiveness, and response to treatment. Yu et al. analyzed the heterogeneity of bladder tumor cells by single-cell transcriptome sequencing. They obtained the data from samples previously uploaded to the Gene Expression Omnibus (GEO) database. With the information of about 12,000 cells analyzed, the authors identified different tumor lineages associated with patient survival. In particular, the EMT (Epithelial-Mesenchymal Transition)-like subtype exhibited the worst prognosis and showed enrichment in pathways involved with cell migration, angiogenesis, and hypoxia (Yu et al.). Although the study requires further validation at the cellular level and with an independent cohort, it shows some interesting lines of research.

Deregulation of N7-methylguanosine (m7G) may contribute to the development of hepatocellular carcinoma. Chen et al. evaluated the expression pattern of regulators of the modification in m7G. The authors used bioinformatic analysis of transcriptome data from hepatocellular carcinoma patients. So, they obtained the data from The Cancer Genome Atlas (TCGA) and GEO databases. After analyzing 29 genes that regulate m7G–modification, the authors established a risk model for hepatocellular carcinoma. This model was generated by selecting seven genes using the data from TCGA. Then, the authors validated their model using the data from GEO. Using the risk signature with these genes, the authors showed they could predict the prognosis for patients with hepatocellular carcinoma (Chen et al.).

Another relevant epigenetic modification is the internal modification of RNA by N6-methyladenosine (m6A). m6A RNA methylation regulates alternative splicing, thus modulating gene expression. Three protein complexes are known to regulate m6A RNA methylation. By using TCGA database, Maimaiti et al. evaluated the clinical role of m6A regulators within the tumor microenvironment in low-grade glioma patients. The authors analyzed the contribution of 12 of these genes, finding that seven were associated with overall survival. Next, they identified m6A-related alternative splicing events. The latter were analyzed using Least Absolute Shrinkage and Selection Operator (LASSO) regression, thus allowing the authors to create a model to predict glioma prognosis. Patients within the high-risk score group presented a higher infiltration score, as determined by TIMER (Tumor Immune Estimation Resource) and CIBERSORT analyses. Remarkably, most tumors having high infiltration scores contained anti-tumoral cells, such as activated NK cells, CD8^+^ T cells, and M1 macrophages (Maimaiti et al.).

On the other hand, Pan et al. also analyzed the prognostic value of members of the chromobox family (CBX) by bioinformatic analysis (Pan et al.). This family is part of polycomb repressive complexes 1, which regulate gene expression. Several members play a role in cancer progression (Lin et al., 2020). Pan et al. compared the transcript expression of CBXs between hepatocarcinoma and normal adjacent tissue. Datasets were obtained from the Gene Expression Profiling Interactive Analysis (GEPEIA). Then, the authors constructed a prognostic model based on the CBX expressions with this information. Their findings show that some CBXs, particularly CBX3, are associated with poor overall survival (Pan et al.). Although the authors found that immune cells upregulate CBX3, and they propose that this CBX modulates the microenvironment *via* immune cells, this hypothesis needs further verification.

RNA-binding proteins (RBPs) are a set of proteins that bind target RNAs thanks to an RNA-binding domain. RBPs can bind to coding and non-coding RNAs, thus regulating different RNA processes. Cancer deregulates some RBPs, but research is needed to test their role in prognosis and therapy. Miao et al. analyzed whether RNPs are differentially expressed in non-small cell lung cancer (NSCLC) and can be prognosis factors. The authors used TCGA, the GEO, and the University of California Santa Cruz (UCSC) Xena data to identify differentially expressed RBPs. Then, they constructed a signature with eight RBPs utilizing LASSO regression analysis, which divided patients into high-risk and low-risk groups. The high-risk group was associated with a more advanced tumor stage and decreased survival. The authors validated the expression of the eight RBPs found in their signature with an independent cohort. They also analyzed the immune infiltration with bioinformatics tools and compared both groups. High-risk patients showed higher infiltration of immune cells with known antitumoral properties (Miao et al.). To verify this contradictory result, further demonstration using immunohistochemistry is needed.

POLE (DNA Polymerase Epsilon, Catalytic Subunit) is a polymerase implicated in DNA replication. Thus, mutations in this enzyme might promote carcinogenesis and be a potential biomarker. Jiang et al. evaluated mutations in POLE using a cohort of patients with colorectal cancer and from TCGA. The authors divided the patients into three groups: those presenting exonuclease domain mutation (EDMs), non-EDMs, and wild type. This study showed that patients with lesser susceptibility to recurrence or progression were within the POLE EDM group. Also, patients from this group with high microsatellite instability have higher overall survival and progression-free survival than patients with tumors with low microsatellite instability and not having POLE mutations Jiang et al.. Nevertheless, patients from TCGA cohort did not present the same behavior, thus highlighting the need for validating bioinformatic studies using independent cohorts besides those obtained from public databases.

Deregulation of the cell cycle has been long known to promote carcinogenesis, but the study of Zhang X et al. show new findings in this field. They analyzed the role of kinesin superfamily member 2C (KIF2C) as a possible prognostic biomarker in cancer. KIF2C participates in spindle formation and sister chromatid separation, among other functions. Zhang X et al. evaluated the correlation of KIF2C expression with several parameters, such as prognosis, tumor mutation burden, and immune infiltration. The authors employed publicly available databases, including TCGA, UALCAN (the University of Alabama at Birmingham Cancer data analysis portal), and TIMER 2.0. They found that KIF2C is upregulated in many types of cancer, which agrees with previous publications (Shimo et al., 2008; An et al., 2021). Immunohistochemistry also corroborated that liver cancer cells highly express KIF2C compared with normal tissue (Zhang X et al.).

MicroRNAs (miRNAs) are small non-coding RNA sequences that regulate mRNA expression. As RNA regulators, they are also implicated in several oncogenic processes but also as tumor suppressors. Li J et al. explored the participation of miR-372-3p as a tumor suppressor in colon cancer. They found that tumor tissue samples downregulate miR-372-3p. In particular, tissues that express low miR-372-3p levels show high expression of the proliferation marker Ki-67. This inverse correlation is also present between miR-372-3p and MAP3K2, which regulates the JNK and ERK5 proliferative pathways. *In vitro* assays showed that miR-372–3p reduces cell proliferation and downregulates MAP3K2 expression. Thus, targeting MAP3K2 or inducing the expression of miR-372–3p might be considered an optional treatment strategy (Li J et al.).

Long non-coding RNAs (lncRNAs) are RNAs with more than 200 nucleotides long that regulate gene expression. These RNAs can be either oncogenic or tumor suppressors; thus, their deregulation favors carcinogenesis. Andonegui-Elguera et al. review the alterations of lncRNAs that lead to genomic instability, showing how two hallmarks are closely intertwined. Consequently, deregulated expression of lncRNAs might lead to resistance to chemotherapy. LncRNAs can regulate cellular processes such as autophagy, which is responsible for recycling organelles and macromolecules (Andonegui-Elguera et al.). Duan et al. analyzed the profile of lncRNAs involved in autophagy in colorectal cancer. The authors distinguished about 13000 lncRNAs from the TCGA database and about 900 from the Human Autophagy database (HADb.) From these data, the authors identified 1342 autophagy-related lncRNAs. Using univariate and multivariate -COX regressions and LASSO regression analysis, the authors selected 11 autophagy-related lncRNAs to classify colorectal cancer patients into two groups per lncRNA: high- and low-risk. High-risk patients showed significantly lower mortality than low-risk patients (Duan et al.). Of note, LINC01011 is upregulated in low-risk colorectal cancer patients. This molecule inhibits mitochondrial fission and increases cisplatin sensitivity in tongue cancer (Fan et al., 2020). As mitochondrial dynamics play a role in chemotherapy sensitivity, this observation deserves further study.

Ferroptosis is a recently identified form of regulated cell death that depends on iron and ROS (Xie et al., 2016). Deregulation of this process may take part in cancer progression. Li Y et al. explored the link between lncRNAs and ferroptosis on the prognosis of pancreatic cancer. This bioinformatics study used data from TCGA and FerrDb databases. Six ferroptosis-related lncRNAs served to construct a signature associated with the survival of pancreatic cancer patients. These lncRNAs were found to participate in the regulation of ROS. In particular, MIR193BHG is associated with autophagy (Li Y et al.). As ROS production and autophagy are processes where the mitochondria are implicated, the ferroptosis-related lncRNAs found by Li et al. might have a role in mitochondria dynamics.

## 3 Epigenetics and anti-tumoral therapies

The search for novel chemotherapeutic drugs is an active research field. Tulsyan et al. review some epigenetic mechanisms deregulated in cancer. Some of these mechanisms are promising not only for finding biomarkers for diagnostic and prognosis but also for developing novel epigenetic drugs (Tulsyan et al.). On the other hand, Contreras-Sanzon et al. review how histone deacetylases (HDACs) participate in several mechanisms of resistance to therapy in lung cancer cells. Besides chromatin remodeling, HDACs regulate other cellular processes; thus, their alteration favors tumor progression. This review shows some preclinical and clinical evidence of the use of HDAC inhibitors to treat different subtypes of lung cancer (Contreras-Sanzon et al.). Mathur et al. focus on the epigenetic mechanisms found in breast cancer, particularly those associated with resistance (Mathur et al.). As in lung cancer, HDAC inhibitors might have some future for treating some breast cancer subtypes.

The study of Zhang Y.Q et al. on quercetin, a compound derived from herbal medicine, shows some novel targets in lung adenocarcinoma. They looked for possible targets with bioinformatics tools (SwissTargetPrediction and Protein Data Bank). The authors also analyzed differentially expressed target genes for quercetin in TCGA and created a risk model for lung adenocarcinoma patients (Zhang Y.Q et al.). The bioinformatics analysis showed that some genes associated with disease prognosis might be targets for quercetin treatment, but this is yet to be confirmed experimentally.

Histone lysine demethylases (KDMs) are enzymes that modulate histone methylation. In particular, the KDM4 subfamily has been considered an attractive target because some members are oncogenic and several tumors overexpress these molecules. Del Moral-Morales et al. screened in the COCONUT (COlleCtion of Open Natural ProdUcTs), the FDA, and DrugBank databases and employed molecular docking to look for potential inhibitors against KDM4 enzymes. Several members of the KDM4 subfamily are upregulated in cancer, as determined by bioinformatics analysis of TCGA. Using this strategy, the study found some potential candidates for therapeutic use and could orient the research of novel treatments (Del Moral-Morales et al.).

In this regard, the study of Fei et al. explored mitochondrial topoisomerase I (TOIP1MT) as a possible target for cancer therapy. TOIP1MT keeps the integrity of mitochondrial DNA (mtDNA) and promotes tumor cell proliferation by regulating several metabolic pathways. Using the GTEX database and several bioinformatics approaches, the authors analyzed the expression of TOP1MT, its prognostic value, and its correlation with immune cells in different types of cancer. Compared with normal tissue, different types of cancer upregulate TOIP1MT, which correlates with poor overall survival, disease-specific survival, and progression-free periods. TOP1MT positively correlates with some immune cells (Fei et al.). This finding deserves further exploration, as this correlation was found with cells known to be antitumoral, such as CD8^+^ T cells and macrophages.

Predicting patients’ responses to anti-cancer treatment is highly desirable. Borutinskaite et al. explored the pattern of genes and proteins from bone marrow samples of acute promyelocytic leukemia (APL) patients undergoing treatment (Borutinskaite et al.). Lactate dehydrogenase (LDHA) takes part in metabolic reprogramming and tumor progression (Romero-Garcia et al., 2016). Mass spectrometry analysis showed that, among others proteins, LDHA levels increase during treatment and at relapse. Also, tumors from patients with no relapse downregulate WT1, CALR, CAV1, and MYC genes (Borutinskaite et al.). Thus, these molecules are promising candidates to track response to treatment in patients with APL.

**FIGURE 1:**
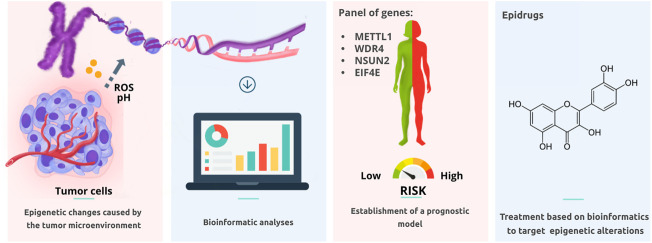
The study of epigenetic alterations and how they have a role in the prognosis and resistance to current therapies will lead to the discovery of novel biomarkers and the elucidation of new treatments.

Although many of these articles were based on bioinformatics and require validation, they provide insights into promising research areas (See Figure 1). As noted, some studies found that certain oncogenic molecules (RPBs, TOIP1MT, KIF2C, among others) are positively correlated with antitumoral immune infiltrate, as evaluated by bioinformatics analysis. Because antitumoral infiltrate has been shown to independently correlate with a good prognosis, further verification at the protein level is highly recommendable. On the other hand, understanding mitochondrial dynamics is relevant for a better knowledge of the evolution and treatment of cancer. The studies contained in this topic might shed some light on this issue.
